# Supplementary material for: Stochastic optimal open-loop control as a theory of force and impedance planning via muscle co-contraction
Source: PLoS Comput Biol. 2020 Feb 28;16(2):e1007414. doi: 10.1371/journal.pcbi.1007414 (PMC7065824; doi:10.1371/journal.pcbi.1007414)
Supplement: Attachment — Submitted filename: response_file3.pdf [file pcbi.1007414.s001.pdf]

Answer: We thank the reviewers for their comments. We provide below a point-by-point response to all the questions they raised. Our answers appear in blue.

Reviewer #1: This paper addresses an important problem: the modelling of feedforward control necessary to deal with large sensory delays in human sensorimotor control i.e. motion planning. Specifically, the paper develops a method to compute the force and mechanical impedance (or muscles activation) for learned arm movements, considering intrinsic motor noise. The method is illustrated by simulating well selected experimental studies from the literature. The presentation is clear and accurate, and i have no major concern. In the following, i will first give general comments, then list minor suggestions.

General comments:

1. The paper seems to target co-contraction of antagonist muscle pairs, however i) mechanical impedance at a joint arises from the co-contraction of the group of all muscles actuating this joint, ii) i guess the technique developed in this paper is valid for such muscle groups, not only for antagonist muscle pairs. If ii) holds, the paper could be formulated in the more general case i).

Answer: It is true that the method works equally well for muscle groups. We often refer to pairs of agonist/antagonist muscles for simplicity but the framework is general enough to handle a group of muscles (as suggested by the simulations with the 6-muscle model). We revised the text accordingly and now refer to group of muscles instead of pairs of agonist/antagonist muscles when relevant.

2. Muscle viscoelasticity varies with their activation, and the human nervous system (NS) can control muscles to shape the interaction with the environment, i.e. impedance control. This environment may involve stable or unstable dynamics, and contain noise. In the paper's current formulation, only internal motor noise is considered, not environmental noise. This should be corrected.

Answer: It is true that we mostly focused on internal motor noise but the noise in the dynamics can also capture environmental noise. We corrected our formulation (in particular in the Author Summary and Lines 41 and 57-58).

3. Impedance control is shaped by muscle mechanics, stretch reflexes as well as long-delay reflexes, see [Franklin2007, Franklin2008]. Long-delay reflexes are missing in the corresponding description at lines 27-31. Note that they still come naturally under feedforward motor commands as defined by the authors as "defined prior to movement execution".

Answer: We modified the text according to the suggestion of the reviewer. We initially considered long-delay reflexes separately in the Introduction because of our considerations of open-loop versus closed-loop control. However, at this point of the Introduction, it is not necessary to make this distinction. We adapted Lines 25-31. Note that we already mentioned Lines 54-55 for instance that feedback gains can be planned and contribute to the feedforward control of impedance, but the actual motor command (if one thinks of the descending signals to the motoneurons) cannot be exactly known in advance because their activation does require knowledge of the current system state (see Lines 55-56).

4. Mechanical impedance is not just stiffness (see e.g. line 33), but can be expressed as corresponding to viscoelasticity. In fact several studies such as [Milner1993] have demonstrated the ability of the NS to adapt wrist viscosity (thus not just stiffness can be controlled, although stiffness and viscosity will co-vary).

Answer: We agree with the reviewer. We just wanted to give an example there. To be more accurate, we now use the term “viscoelasticity” to illustrate the notion of mechanical impedance. See Abstract and Author Summary in particular, and Lines 267-269.

5. To facilitate the reading, would suggest adding a figure 1 to describe the different setups corresponding to equations 1, 14-16, close to these equations and with the related parameters.
Answer: We thank the reviewer for the suggestion. However, we believe that the model in Eq. 1 is very simplified and that an illustration would not be especially informative for the reader. The model in Eq. 16 (Katayama & Kawato) is in contrast quite involved and we only briefly describe it in the text. To better understand the details of this model, we believe the reader should look at the paper of Katayama and Kawato where an illustration is provided together with full details about the notations.

6. The proposed model can predict muscles activation of movements learned in various stable and unstable environments. Similarly, the model of [Franklin2008,Tee2010] can predict at least the experiments of figures 1,2,4,5. It is currently just mentioned at line 523, but i think a comparison with the new model should be provided. In my understanding:
- The new model determines the trajectory, force and impedance corresponding to the learned behaviour of a limb with known kinematics and dynamics, in a known dynamical environment.
- On the other hand, the model of [Franklin2008,Tee2010] can learn the force and impedance along a reference trajectory, and does not need a-priori knowledge of the plant and environment dynamics or kinematics. Note that while model is formulated in an ad-hoc way in [Franklin2008,Tee2010], it in fact corresponds to the gradient descent minimisation of error and effort as analysed in [Yang2011].
- I guess that the simulations of reaching forward movements with lateral instability, the conditions are different from the experiment [Burdet2001] and the simulations of [Franklin2008,Tee2010], where the external force drops when the hand deviates laterally more than x centimeters from the straight line (the experiment would be dangerous and tiring to carry out without this). This may explain the different terminal muscle activation in the simulations in Fig.4,5.
Answer: We added more elements regarding the comparison with these previous models (see Lines 535-545 in Discussion). For our simulations, indeed, we did not remove the divergent force field when the hand deviated more than x centimeters from the straight line. We performed simulations by implementing this force field removal and results were actually very similar to the ones presented in the paper.
Actually, the muscle activations in these simulations seem to depend more on the cost function design. For instance, one may adjust the weight of the variance cost. One could also introduce a running variance cost etc., but we did not test extensively all these possible variants of modeling in the present paper. Finally, note that the depicted muscle activations also depend on the specific 6-muscle model under consideration in our study (other muscle models could yield different optimal muscle activations).

7. As pointed out by the authors, the major difference of their model to SOC is that it is feedforward while SOC is "closed-loop". I would stress this difference even more by calling the SOC closed-loop control at every opportunity in the text. (also it would be possible to use SOC corresponding to the control community which invented it rather than SOFC used much later in the computational neuroscience community, and FSOC (i.e. feedforward SOC) could then be used instead of SOOC?)
Answer: In this revision, we tried to emphasize the closed-loop aspect of SOC more systematically. We now use SOC instead of SOFC. Regarding the acronym of the proposed framework, we would prefer to keep using SOOC because we also used this term in a companion paper. Hence, for the sake of coherence, we believe it is better to have the same terminology.

Minor suggestions:
Answer: We thank the reviewer for all the suggestions provided below, which greatly improve the accuracy of our paper.

Abstract
While these approaches have yielded valuable insights about motor control, they typically fail explain a common phenomenon known as muscle co-contraction. Co-contraction of agonist and antagonist muscles contributes to modulate the mechanical impedance of the neuromusculoskeletal system (e.g. joint stiffness) and is thought to be mainly under the influence of descending signals from the brain.
->
While these approaches have yielded valuable insights about motor control, they typically fail in explaining muscle co-contraction. Co-contraction of a group of muscles associated to a motor function (e.g. agonist and antagonist muscles spanning a joint) contributes to modulate the mechanical impedance of the neuromusculoskeletal system (e.g. joint viscoelasticity) and is thought to be mainly under the influence of descending signals from the brain.
Answer: Changed.

Optimal feedback (closed-loop) control, preprogramming feedback gains but requiring on-line state estimation processes through long-latency sensory feedback loops,
->
Optimal closed-loop control, ...
Answer: Changed.

Author summary
to explain the planning of force and impedance (e.g. stiffness)
->
to explain the planning of force and impedance (e.g. viscoelasticity)
Answer: Changed.

A major outcome of this mathematical framework is the explanation of a long-known phenomenon called muscle co-contraction (i.e. the concurrent contraction of opposing muscles).
->
A major outcome of this mathematical framework is the explanation of muscle co-contraction (i.e. the concurrent contraction of a group of muscles involved in a motor function).
Answer: Changed.

line 9
On the other hand, stochastic optimal –feedback– control (SOC or SOFC) theory was developed to account for the
->
On the other hand, stochastic optimal control (SOC) was used to account for the ...
Answer: Changed.

16
The SOFC theory led to a number of valuable predictions among which the minimal intervention principle, stating that errors are corrected on-line only when they affect the goal of the task, is a significant outcome [9].
->
The SOC theory led to a number of valuable predictions among which the minimal intervention principle,

stating that errors are corrected on-line only when they affect the goal of the task [9].
Answer: Changed.

19
However, these two prominent approaches have in common that they fail to simply account for a fundamental motor control strategy used by the central nervous system
->
However, both of these approaches fail at accounting for a fundamental motor control strategy ...
Answer: Changed.

21
co-contraction or co-activation of antagonist muscles
->
co-contraction or co-activation of muscles groups
Answer: Changed.

27
This effect does not only result from the summation of intrinsic stiffnesses of opposing muscles [20, 21] but also from nonlinear stretch reflex interaction [22, 23].
->
This effect results both from the summation of intrinsic stiffness of muscles around a common joint [20, 21] and reflexes [22,23,Franklin2007].
Answer: Changed.

33
First, co-contraction contributes to modulate the effective limb's impedance (e.g. joint stiffness),
->
... the effective limb's impedance (e.g. joint viscoelasticity),
Answer: Changed.

48
More fundamentally, an optimal feedback control scheme requires
->
More fundamentally, a closed-loop optimal control scheme requires ...
Answer: Changed.

53
This may seem to contrast with the feedforward nature of impedance and co-contraction control that has been stressed in several studies [16, 18, 34–36].
->
This may seem to contrast with the feedforward nature of impedance and co-contraction control [16,18,34,Franklin2013B]
(xxx these papers present direct experimental evidence for the feedforward nature of impedance control)
Answer: Changed.

57
As this ability may be limited in some cases (e.g. unstable task or too fast motion), co-contraction

->
As this ability is limited in some cases (e.g. unpredictable interaction with the environment, unstable task or too fast motion), co-contraction
Answer: Changed.

82
Although we use the term open-loop –in the sense of control theory–we do not necessarily exclude the role of automatic short-latency reflexes that contribute to the spring-like behavior of intact muscles beyond their short-range stiffness.
->
... we do not necessarily exclude the role of reflexes that contribute to the spring-like behavior of intact muscles beyond their short-range stiffness.
Answer: Changed.

90
Our working hypothesis is that both force and impedance are planned
->
Our working hypothesis is that both force and mechanical impedance are planned
Answer: Changed.

93
open-loop controls
->
open-loop control
Answer: Changed.

128
where R, Q and Qf are positive definite and positive semi-definite matrices with appropriate dimensions respectively.
->
where R, Q are positive definite matrices and and Qf is a positive semi-definite matrix, all of appropriate dimensions.
Answer: Changed (but note that Q and Qf were the positive semi-definite matrices).

130
it can be put out of the expectation
->
it can be taken outside the expectation value integral
Answer: Changed. “Because u(.) is a deterministic function by hypothesis, the related integral value can be taken outside the expectation operator”.

145
has a nonlinear dynamics
->
has nonlinear dynamics
Answer: Changed.

152
in agreement with the well-known minimum variance model
->
in agreement with the minimum variance model
Answer: Changed.

173
1st order Taylor approximations
->
first order Taylor approximations
Answer: Changed.

lines 200 to 220:
is it necessary to invoke Feldman (thus a physiological hypothesis) here, or would a linearisation do the same job
Answer: If one linearizes a nonlinear system using standard methods, then one should obtain a linear system and not a nonlinear system like in Eq. 9. Actually, a “statistical linearization” is needed to capture the relevant nonlinear effects to exploit co-contraction and impedance. Eq. 9 is a nonlinear system that is necessary to have a control on the variance via impedance regulation. Invoking Feldman’s work is not strictly necessary at this point but it may be a good reference to interpret this model. We slightly adjusted the related sentence to improve our purpose.

230
To illustrate an enlightening point, let us focus on horizontal movements now. The system then simplifies as follows:
->
Focusing on horizontal movements, the system then simplifies to:
Answer: Changed.

268
A two degrees-of-freedom (dof) version of the arm with 6 muscles was also considered to simulate planar arm reaching movements, corresponding to the full model of [51].
->
A two degrees-of-freedom (dof) version of the arm with 6 muscles was also considered to simulate planar arm reaching movements. This is exactly the full model described in [51].
Answer: Changed.

273
C is the Coriolis/centripetal term
->
C \dot{q} is the Coriolis/centripetal term
Answer: Changed.

276
The net joint torque vector was a function
->?

The net joint torque vector is a function
Answer: Changed.

291
In the seminal study of Hogan described above [46],
->
In Hogan's study [46] described above,
Answer: Changed.

302:
i) add: "where the parameters are defined by Eq.1."
ii) what are the units of the parameters in this simulation?
Answer: (i) Added. (ii) Units were indicated when relevant. Note that some parameters are dimensionless (T and K for instance). The matrices R and Q, Q_f are also dimensionless. Full details about the parameters of the simulations are now given in the caption of Figure 1.

309
in such an unstable posture
->
in the unstable posture
Answer: Changed.

311
(remind that we prevent feedback control)
->
(remind that feedback control is prevented)

314
In the loaded case, the task instability is increased
-> what does this mean? what would be the measure of stability?
Answer: Changed. “In the loaded case, the destabilizing gravity torque is increased and optimal co-contraction levels become larger to counteract it”

Fig.1
the lines are currently hardly visible. To increase the visibility, one could e.g. reduce the position range to e.g. [-3,3] and the velocity range to e.g. [-10,10], and indicate the standard deviation using e.g. a fine dotted line?
Answer: We improved Fig. 1 as suggested.

348
in order to model that co-contraction does not lead to increased variability
->
in order to model that co-contraction does not lead to increased variability [Burdet2001]
Answer: Changed

Fig.2 For these simulations, why using different q_var values? could for example all simulations be done with q_var=5000 ?

Answer: Yes, we can perform all the simulations with the same q_var. The Figure has been modified.

362
behavior of subjects described in [61]
->
behavior of subjects in this experiment
Answer: Changed.

Fig.3, similar to Fig.1, could the visibility be improved?
Answer: It is hard to better rescale the graphs here like in Fig. 1. However, we can increase the width of this Figure for better visibility.

397
Therefore, it was impossible for the subjects to predict
->
Because the hand would start with random lateral deviation due to motor noise, it was not possible for the subjects to predict ...
Answer: Changed

404
(e.g. participants kept co-contracting when the divergent force field was unexpectedly removed) [18, 65–67].
->
[18, Franklin2003B,66]
Answer: Changed.

418
there was a paper by Wolpert around 1996 showing how the visual feedback can lead to deforming the hand trajectory, which may back the use of a jerk term for this simulation
Answer: We believe the reviewer refers to this paper "D. M. Wolpert, Z. Ghahramani, and M. I. Jordan, "Are arm trajectories planned in kinematic or dynamic coordinates? An adaptation study.", Exp. Brain Res. 103, 3 (1995), pp. 460—470". We added this reference to justify the use of the jerk here.

445
We noticed this is actually a limit of the 6-muscle model used in these simulations, which does not allow arbitrary geometries for the endpoint stiffness in a given posture
->
xxx note that the geometry of the 2 link model allows modifying the stiffness ellipse shape and orientation, see e.g. [Tee2010]
similarly the difference to [Tee2010] could be mentioned in lines 450-460.
Answer: We agree, which is why we insisted on the fact the "for this posture". We modified the text to be more accurate. We also added a sentence to mention that other muscle models could yield stiffness ellipses elongated in the direction of instability. See Lines 455-465.

462
Finally, we revisit the minimum intervention principle [9]. This well-known principle is most simply illustrated in a pointing-to-a-line task as in [9, 69, 70].

->
Finally, we revisit the minimum intervention principle [9] as illustrated in a pointing-to-a-line task in [9, 69, 70].
Answer: Changed

502
the consideration of open-loop controls
->
the consideration of open-loop control, ...
Answer: Changed

516
effort and energy-like criteria are often minimized in optimal control models which tends to prevent
->
... which tend to prevent
Answer: Changed.

523
Researchers have nevertheless attempted to explain co-contraction or its contribution to impedance in existing DOC or SOFC frameworks, but this was often an ad-hoc modeling [75,76].
->
xxx i believe this requires more discussion, and a comparison with this paper's results. To note, [75, Franklin2008] are formulated in an ad-hoc way, but the underlying mathematical principle is described in [Yang2011].
Answer: We added some sentences in the Discussion to better stress the main differences with these papers. See Lines 535-545.

[Milner1993] TE Milner and C Cloutier C (1993), Compensation for mechanically unstable loading in voluntary wrist movement. Experimental Brain Research 94(3): 522-32.

[Franklin2007]
DW Franklin, G Liaw, TE Milner, R Osu, E Burdet and Kawato (2007), Endpoint stiffness of the arm is directionally tuned to instability in the environment. Journal of Neuroscience 27(29): 7705-16.

[Franklin2008]
DW Franklin, E Burdet E, KP Tee, R Osu, CM Chew, TE Milner and M Kawato (2008), CNS learns stable, accurate, and efficient movements using a simple algorithm. Journal of Neuroscience 28(44): 11165-73.

[Franklin2003B]
DW Franklin, E Burdet, R Osu, M Kawato, TE Milner (2003), Functional significance of stiffness in adaptation of multijoint arm movements to stable and unstable dynamics. Experimental Brain Research 151(2): 145-57.

[Tee2010]
KP Tee, DW Franklin, M Kawato, TE Milner and E Burdet (2010), Concurrent adaptation of force and impedance in the redundant muscle system. Biological Cybernetics 102(1): 31-44.

[Yang2011]
C Yang, G Ganesh, S Haddadin, S Parusel, A Albu-Schaeffer and E Burdet (2011), Human-like adaptation of force and impedance in stable and unstable interactions. IEEE Transactions on Robotics 27(5): 918-30.

Reviewer #2: This paper proposes a stochastic optimal open-loop control theory which enable to plan the movement and the stiffness of a biological system moved by antagonistic muscles. The core of the work is to show how such a model is able to easily exploit the use of co-contraction to account for task uncertainties/disturbances.

The idea is very interesting, and the paper presents a novel contribution which is worth for consideration. However, I have several comments that I would ask the authors to consider as suggestions to improve their manuscript.

The major drawback that I see in this work is related to the significant variability in the definition of cost functions to implement the approach in the different experimental conditions. I am pretty convinced that there could be a unique definition of a general problem definition/cost function able to "work" in all the conditions. This is also motivated by a biological counterpart; indeed it is pretty unlikely that the human motor control employ different feedforward strategies for different tasks, but rather I would expect an unifying framework (which is one of the main point of strength for the equilibrium point hypothesis). I think that this aspect is at least worth a discussion in the manuscript, together with a clarification on the particular choices in defining the optimisation problems.

Answer: We thank the reviewer for his comments. We agree that it would be nice to have a single cost function to replicate all the experimental findings of the present paper. Actually, there is a general picture that emerges from the present paper since we always consider a trade-off between an "effort" term and a "variance" term (these terms are listed as critical ingredients to get co-contraction patterns, see Line 509-512 as well as in the Abstract). However, the variety of models (e.g. choice of coordinates) and tasks under consideration makes it impossible to define a unique cost function throughout the whole study. For example, effort can be written differently depending on whether muscles are modeled or not, and variance can be expressed in joint space or Cartesian space etc. Our approach was rather to show the versatility of the SOOC framework to handle a variety of tasks and models.

Additional comments are provided below, divided in major and minors.

Majors:
- In Fig. 1A, the plot of variance in position and velocity plots is not visible, maybe the authors could try with a different set of colors.
Answer: This was also noted by Reviewer #1 and we have changed the scale to better visualize the graphs.
- The description of Fig.1 is a bit confused, I would suggest naming the 4 subplots of subfig A (and the same for B) and refer to those labels in the caption in an ordered way.
Answer: We improved the figure by adding labels and rewriting the text of the caption.
- I would expect that, as soon as an equilibrium is reached, the parameters are maintained constant for the whole execution. In the simulations shown in Fig.1, instead, it seems that the optimal solution shows some oscillations in the last 0.5 seconds. Is there a modeling reason for this, or it is related to the optimization itself? I think this is a relevant aspect to discuss.
Answer: The oscillations in the trajectory and control reflect both boundary conditions, the terminal cost and the finite motion duration. The initial change arises because the initial state of the plant is not optimal, and the control must move the system to a better state. The final changes rather come from the terminal cost Qf and the fact that we have a finite time horizon in our simulations. If we set Qf to zero for instance, the muscle torques would decrease to zero at the final time. If we extend the time horizon, the middle

steady-state phase would extend as well. We added a few words about these considerations to help the reader (see Lines 321-325).

- In section “Reaching task with the forearm” the authors refer to Fig. 2C to show the effect of trajectory-time on the resulting stiffness. However, this is not completely clear in the figure. I guess the higher trajectory-time the lower overall torque, but explicitly indicating the time dependency of the stiffness would be beneficial. Moreover, it could be really interesting observing and comparing the whole optimal stiffness profile at different trajectory-time values (with a suitable time-scaling to enable the comparison).
Answer: We initially reproduced this plot to compare to a Figure in ref [54]. We now also display the stiffness profiles for the different conditions for the sake of completeness.
- Also, in the simulations of section “Reaching task with the forearm” I observe an oscillation in the optimal impedance at the beginning and at the end of the task, is there a “methodological” reason for this? I would expect instead a steady value (as shown in the reference [54] for humans)
Answer: The results should rather be compared to reference [59] where time-varying stiffness profiles, quite similar to the depicted ones, were found.
- Fig 4, the plot of velocities is not clear. Please report them in dedicated subfigures. Controls and Muscles Tensions subfigures are not explained in the caption.
Answer: We apologize for the lack of clarity. The point is that the figure is relatively big and we have to save space (adding a new column of subplots would be make other plots less clear). Hence, we decided to add more information about the axes of the velocity profiles to clarify these subplots. Also, the controls and tensions subfigures are now better described in the caption.
- I would include further discussions regarding the following points:
o what happens if the stiffness cartesian matrix is not diagonal?
Answer: To check this point, we performed new simulations by considering a general positive definite stiffness matrix instead of a diagonal one, and the results were the same than those presented in the paper. We modified the text accordingly because the “diagonal” matrix assumption was not necessary.
o what happens if there is an unpredicted interaction with the environment (e.g. a contact with the environment, thus a force in a specific direction)?
Answer: Contact with the environment can be considered and noise can model some degree of uncertainty about the external force applied by the environment onto the human system. The case of a totally unpredictable interaction with the environment (e.g. no contact force at all or significant contact force on successive trials) would require a different modeling because such an uncertainty cannot be modeled as a Brownian motion (it is rather a structural uncertainty in the dynamics). However, we keep this type of issue for future work.
o Does this approach scale well with the dimensionality of the problem? E.g. is it possible to generalize to full upper limb models?
Answer: Our approach scales relatively well with the dimensionality of the problem (compared to classical SOC) but the state augmentation resulting from the inclusion of the covariance matrix as a part of the state vector may increase the dimensionality of the problem. For a 7-dof arm and a torque control case for instance, the mean part of the augmented state would be of dimension n=14 (position+velocity) and the covariance part would be of dimension (n*(n+1)/2)=105… Hence the total dimension of the state in the optimal control problem would be 119. With existing optimal control software, this may be something that could be handled numerically as nonlinear programming softwares can handle optimization in large spaces. In any case, this should still be much faster than trying to fully resolve a SOC problem in dimension 14 using the HJB formalism for example.
o is it possible to model dual arm constraints (e.g. executing a task while holding an object)?

Answer: The method applies to any system or problem that can be modeled as a controlled Ito stochastic differential equation with costs and constraints on the mean and covariance of the stochastic state process. This is quite general. Hence, it should be relevant to handle constraints like those needed to model holding an object while executing a task.

Minors and Typos:
- In abstract: “fail to explain”
Answer: Corrected.
- In abstract, the sentence “Optimal feedback (closed loop) control, preprogramming feedback gains but requiring on-line state estimation processes through long-latency sensory feedback loops, may then complement this nominal feedforward motor command to fully determine the limb’s mechanical impedance.” Is too long, I would suggest to rephrase by splitting in two.
Answer: Corrected according to the advice of Reviewer #1
- In caption of Fig. 1A, “corresponding individual muscle torques are depicted below (black for the flexor activation and gray for the extensor activation)” shouldn’t be filled and dashed line instead?
Answer: We apologize for the confused caption. The reviewer is correct. We clarified the text in this caption as suggested above.
- Line 210 --- kd = sqrt(iks)? Is not 1/2
Answer: We used the definition of the damping ratio as (actual damping)/(critical damping). Here, the critical damping was 2*sqrt(iks), hence the 1/2 result.
- Line 225 --- Weight factors α, β and qvar can be chosen to adjust the optimal behavior of the system. How do you select these parameters?
Answer: This is true that the design of the cost function will affect the optimal solution. This is typical of the optimal control framework more generally. Here these factors have the following meaning. The factor α is the weight of ‘co-contraction’ in the effort term (with respect to the cost of net torque) and qvar is the overall weight of the variance term. The role of β is perhaps more minor as it is only used to dissociate the position-related variance and the speed-related variance in the state vector. We renamed β as qv to have a better notation and because beta is used also in the paper to set the magnitude of the divergent force field. Note that in our simulations, we did not try to carefully adjust these weights (for instance we just took α=1, but we tried to select a variance weight such that the magnitude of the predicted stiffness was comparable to experimental data (as a rule of thumb, a small qvar would lead to low stiffness –zero in the limit– while a large qvar would lead to high stiffness). As our model is based on a compromise between effort and variance, at least one parameter must be adjusted to determine the optimal behavior (e.g. increase effort to reduce variance or the other way around).
- Eq 26 should be followed by a comma and not a dot
Answer: Thanks. Corrected.

Reviewer #3: In the article, the authors use an Optimal Control framework to develop multiple models in different state-spaces (muscle level and joint level) to show that the optimal control principles can be used to explain the co-contraction in human movements. This is an important attempt, as the modelling of co-contraction has not been done in this framework before, and the simulation results have replicated multiple experimental results.
Although this is a very interesting approach I have several concerns.

Major.
The key idea in the joint level model is that there is a reference trajectory, which is controlled by joint torque, and the deviation from this trajectory, which is controlled by the co-contraction. The two parts are separable. In other words, there is the part of the model that deal with the trajectory planning, which is not new when it comes to modelling. This planned trajectory is subjected to system noise, and therefore there is another, one-input controller, where the only control input is stiffness. Is this then a non-trivial result, that such model predicts stiffness control? Would the results still hold, if the control was dependent on the torque too?
Answer: This joint-level model is used for the purpose of illustrating how stiffness control may arise in the proposed framework and for illustrating this theoretical uncoupling of net torque control and stiffness control. However, in more general cases (e.g. a planar 2-dof arm or a 1-dof arm with gravity torque), this result would not hold. However, the same theoretical derivation can still be applied. This was said Line 228 and Lines 243-246.

Inferring from Fig. 1, numerically the state x and control u are of the similar order of magnitude. However, the selected costs for that model Q and R differ by 3 or 4 orders of magnitude (line 303). This would mean that the effect of activation cost is small compared to the state dependent costs, suggesting that the co-contraction is energetically cheap, which is not the case in humans. Is this correct? What is the relative weighting of these costs? How sensitive are the results to this cost? Normally, the cost parameters are selected so that the effects of the separate modalities are comparable, otherwise why have it as a cost in the first place?
Answer: In Figure 1, we plotted angles in degrees but in the mathematical model they would be expressed in radians. Hence the order of magnitude is not really similar (x has values much smaller than u actually; typically, x is <0.05 radian while u>1 Nm). Given that these values are squared and integrated over time, this means that there are several orders of magnitude of difference between the variance magnitude and the effort magnitude. Hence, the weights for R and Q which are indeed adapted to make the two cost components (effort and variance) of comparable magnitude. Regarding the sensitivity of the results with respect to the weights of the cost, this is not very sensitive when the weights are similar orders of magnitude. A fine-tuning of the weights was not needed in these simulations.

Specific.
The joint-level model description considers the model as open-loop. However, the behaviour contains the corrections from the reference trajectory, which is clearly feedback control. The authors should clarify what they mean by open loop in this case?
Answer: We mean that the control is open-loop here because both the net torque and stiffness are specified prior to movement execution as functions of time [tau(t) and kappa(t)]. From a control theory terminology this is therefore “open-loop” and there is no feedback at all (i.e. we do not need to estimate the system’s state during execution to correct deviations from the reference trajectory). This is possible because we model the viscoelastic (or spring-like) properties of the muscles (hence this is possible just like a spring can “correct” a deviation without position sensor). From a biological perspective, this means that

the system should be at least able to modify its intrinsic impedance in a feedforward manner (which we assume is in part the role of co-contraction).

Joint level modelling is clearly described and easy to follow. However, the muscle level modelling lacks clarity in definition. Authors provide the equations for the mechanical model behaviour, but the implementation of the controller (at least to me) is unclear and not nearly at the level of the joint level model.
Answer: The model was described more briefly because it was taken from another paper (Katayama and Kawato's paper). We added a few sentences to stress the important points in this model but refer the reader to the original paper by Katayama and Kawato for full details. See Lines 267-269.

I understand that it is probably beyond the scope of the paper, but I would like to see (at least the discussion) of how such model would extend to the case where the feedback control is available. Would co-contraction still be present?
Answer: This is a very good point and we would like to investigate this aspect in the future. The easy way that we mention in the paper would be to use SOOC and then perform feedback control on the top of this nominal feedforward command. However, this method is sequential, meaning that the level of co-contraction cannot depend on whether feedback control would not be a more efficient strategy. Actually, merging the SOOC and SOC approaches in a unique framework considering both control modes at once (and modeling sensory delays for instance) would to be an interesting problem for future work.

The 6-muscle model is unable to learn to increase the endpoint stiffness only in the direction of the instability (Figure 5A). However previous muscle-based models have shown that this is possible when considering costs of stability, accuracy and metabolic cost (Franklin et al., J Neuroscience, 2008; Tee et al., Biological Cybernetics, 2010; Kadiallah et al., PLoS ONE, 2012). Is this because of the specific parameters of the muscle/joint model that was used, or specific to the newly developed SOOC model. Could this result from the extreme low cost of co-contraction such that learning the specific endpoint impedance is not necessary/optimal?
Answer: It is likely that other muscle models may have been used to change the shape of the stiffness ellipse (as mentioned in the referred works). Here we used the model of Katayama and Kawato (with exactly their parameters). When checking all the possible orientations of the stiffness ellipse in the considered posture, we could not find any muscle activation vector that would make the ellipse horizontal (but note that this was possible for other arm postures). We added a few more words on this issue (Lines 450-460). Finally, please note that co-contraction is always a costly strategy in our models, and it is not negligible compared to the variance cost (thanks to the weights in the cost function).
