# Supplementary material for: Stochastic optimal open-loop control as a theory of force and impedance planning via muscle co-contraction
Source: PLoS Comput Biol. 2020 Feb 28;16(2):e1007414. doi: 10.1371/journal.pcbi.1007414 (PMC7065824; doi:10.1371/journal.pcbi.1007414)
Supplement: Attachment — Submitted filename: response_file.docx [file pcbi.1007414.s002.docx]

We thank the editors and reviewers for their comments. Please find below our answers to each question raised.

**Reviewer #1**: The paper is fine to me and the authors have addressed the previous comments in a suitable way.
Three minor points could be addressed:

1. The response had:
C \dot{q} is the Coriolis/centripetal term
Answer: Changed.
however at line 275 i still read "C is the Coriolis/centripetal term"
i do not know whether this is an overlook.
Answer: It was a misunderstanding of the original comment. It is now corrected.

2. Reference [47] seems to be incomplete:
47. Berret B, Jean F. Efficient computation of optimal open-loop controls for stochastic systems; 2019.
Answer: we added details about the reference to this preprint.

3. In the discussion at lines 570-605, if i understand correctly, mechanical impedance is still ascribed to intrinsic mechanical properties and spinal reflexes. However in the studies of Franklin et al. 2007, 2008 etc. mechanical impedance seems to stem also from long delay reflexes.
Answer: To be clearer, we added in the Discussion, L. 604-606: “*As mentioned earlier, mechanical impedance can also be modified via transcortical reflex loops relying on pre-determined feedback gains. However, the associated mechanisms are of a different nature in that they require evolved state estimation processes.*”.

**Reviewer #2**: The authors addressed all the comments I had on the first version of the manuscript and, I believe, the final version of this work is suitable for publication.

**Reviewer #3**: The authors have revised the paper and answered several of my concerns, however I feel that three of these issues are not sufficiently addressed in the current revision.

Q1:
Original: The key idea in the joint level model is that there is a reference trajectory, which is controlled by joint torque, and the deviation from this trajectory, which is controlled by the co-contraction. The two parts are separable. In other words, there is the part of the model that deal with the trajectory planning, which is not new when it comes to modelling. This planned trajectory is subjected to system noise, and therefore there is another, one-input controller, where the only control input is stiffness. Is this then a non-trivial result, that such model predicts stiffness control? Would the results still hold, if the control was dependent on the torque too?
Answer: This joint-level model is used for the purpose of illustrating how stiffness control may arise in the proposed framework and for illustrating this theoretical uncoupling of net torque control and stiffness control. However, in more general cases (e.g. a planar 2-dof arm or a 1-dof arm with gravity torque), this result would not hold. However, the same theoretical derivation can still be applied. This was said Line 228 and Lines 243-246.
Response: If this result does not hold for a realistic case where there is gravity then how general is this solution? This feels like an important point that is glossed over in the current manuscript.

Answer: We presented this simplified scenario to explain the functioning of the joint level modeling. These basic derivations are provided because they allow us to show a result that we think elegant and enlightening (the decoupling in two sub-problems). However, this is in no way a restriction of our approach to this specific case. It does not mean that we cannot find optimal solutions for more realistic scenarios. In fact, we can address more general scenarios (for example with gravity) by using the SOOC framework (this is in particular explained in the subsection entitled “*Stochastic optimal open-loop control for general nonlinear systems*”, p. 7-8). Finally, it is worth noting that the Cartesian model of the planar reaching task in a divergent force field (based on the same type of modeling) also leads to a decoupling in two sub-problems, thereby showing that this specific case can be relevant to other scenarios (see Q3).

We modified Lines 230-247 to clarify our purpose, in particular: “*These derivations are useful to understand the functioning of the model but, in more realistic scenarios, the above decoupling will not hold anymore. This is in no way an issue or limitation because more general cases can be easily handled within the SOOC framework. For instance, if gravity is not neglected, Eq. 11 can be used together with Eq.12 to formulate a nonlinear SOOC problem that can be resolved with the methods described in the previous subsections.*”

Q2:
Original: Inferring from Fig. 1, numerically the state x and control u are of the similar order of magnitude. However, the selected costs for that model Q and R differ by 3 or 4 orders of magnitude (line 303). This would mean that the effect of activation cost is small compared to the state dependent costs, suggesting that the cocontraction is energetically cheap, which is not the case in humans. Is this correct? What is the relative weighting of these costs? How sensitive are the results to this cost? Normally, the cost parameters are selected so that the effects of the separate modalities are comparable, otherwise why have it as a cost in the first place?
Answer: In Figure 1, we plotted angles in degrees but in the mathematical model they would be expressed in radians. Hence the order of magnitude is not really similar (x has values much smaller than u actually; typically, x is <0.05 radian while u>1 Nm). Given that these values are squared and integrated over time, this means that there are several orders of magnitude of difference between the variance magnitude and the effort magnitude. Hence, the weights for R and Q which are indeed adapted to make the two cost components (effort and variance) of comparable magnitude. Regarding the sensitivity of the results with respect to the weights of the cost, this is not very sensitive when the weights are similar orders of magnitude. A fine-tuning of the weights was not needed in these simulations.
Response: The authors response to this is clear, however it would be helpful to mention in the paper that these two cost components are of similar magnitude. As well, as the authors mention that the modelling is done in radians, Figure 1 could also use radians for easier comparison.
Answer: L. 307-308, we added: “*These weights were chosen such as to make effort and variance costs of comparable magnitude (note that angles are in radians).*”. For the figure, however, we believe that providing plots in degrees is more intuitive for most readers (the fact that plots are in degrees is now clearly mentioned in the caption of Fig. 1).

Q3
Original: The 6-muscle model is unable to learn to increase the endpoint stiffness only in the direction of the instability (Figure 5A). However previous muscle-based models have shown that this is possible when considering costs of stability, accuracy and metabolic cost (Franklin et al., J Neuroscience, 2008; Tee et al., Biological Cybernetics, 2010; Kadiallah et al., PLoS ONE, 2012). Is this because of the specific parameters of the muscle/joint model that was used, or specific to the newly developed SOOC model. Could this result from the extreme low cost of co-contraction such that learning the specific endpoint impedance is not necessary/optimal?
Answer: It is likely that other muscle models may have been used to change the shape of the stiffness ellipse (as mentioned in the referred works). Here we used the model of Katayama and Kawato (with exactly their parameters). When checking all the possible orientations of the stiffness ellipse in the considered posture, we could not find any muscle activation vector that would make the ellipse horizontal (but note that this was possible for other arm postures). We added a few more words on this issue (Lines 450-460). Finally, please note that co-contraction is always a costly strategy in our models, and it is not negligible compared to the variance cost (thanks to the weights in the cost function).
Response: This still does not address my concern. Is the SOOC model unable to find a solution that matches human subjects, or is it the feature of the kinematic model used. If the authors could implement this SOOC approach on a model that was already shown to produce this effect, we would know where this limitation arises. The fact that the kinematic model can’t produce this result does not show that the SOOC model can produce this. I realize that the Cartesian mass-point model is a partial approach but still does not fully address the issue.

Answer: We indeed developed the Cartesian mass-point model to show that this limitation arises from the 6-muscle model (and mid-path arm geometry) and not from the SOOC framework itself. The Cartesian model clearly shows that the SOOC framework does adapt the shape of the stiffness ellipse according to the direction of the divergent force field. Moreover, to clearly confirm that the failure to get horizontal stiffness ellipses is due to the underlying musculoskeletal model, we performed an additional verification. We tested all possible activations of the 6 muscles and found that no activation vector could yield horizontal stiffness ellipses at mid-path: hence, it is normal that the SOOC algorithm cannot find this type of solution. Taken together, these results indicate that it is not an issue with the SOOC framework but an issue with the underlying arm model. See our description Lines 452-473:

“*However, while endpoint stiffness increased along the direction of instability, we also found that it increased in the direction of the movement. As such, the stiffness geometry was not really shaped according to the direction of the destabilizing force (see Fig.5A). We found this is actually a limitation of the underlying musculoskeletal model used in these simulations, which does not allow arbitrary geometries of the endpoint stiffness for the current arm posture –the orientation of the depicted ellipses was actually the most horizontal that one can get from this model given the Jacobian matrix at the midpoint of the trajectory–. This was tested by considering all possible muscle activation vectors u and checking that the resultant stiffness ellipse was never oriented horizontally. To increase the stiffness along the x-axis, the algorithm thus had to increase the endpoint stiffness as a whole and not as selectively as in the data of [16,13] (but see [71] in static tasks). Note that this observation does not preclude alternative musculoskeletal models from yielding stiffness ellipses elongated in the direction of instability (e.g. see [70,73]). Instead of considering alternative muscle models, we investigated whether the selective tuning of stiffness geometry could be predicted by the SOOC framework by considering a simpler Cartesian model of the task (following the derivations of Eqs.9-13 but for a planar mass-point system). In this Cartesian model, the cost was only composed of effort and endpoint variance terms (no smoothness term was needed because optimal paths were straight when minimizing effort). Using this Cartesian model, it becomes clear that the optimal endpoint stiffness predicted by the SOOC framework is shaped according to the direction of the destabilizing force (see Fig.5B). The interest of this Cartesian model is to show that the change of stiffness geometry in the divergent force field can be explained within the SOOC framework. The interest of the muscle model was to show that muscle co-contraction may indeed underlie the increase of endpoint stiffness.*”.
